# A Novel Strategy for Improving the Aeromagnetic Compensation Performance of Helicopters

**DOI:** 10.3390/s18061846

**Published:** 2018-06-06

**Authors:** Luzhao Chen, Peilin Wu, Wanhua Zhu, Yongqiang Feng, Guangyou Fang

**Affiliations:** 1 Key Laboratory of Electromagnetic Radiation and Sensing Technology, Chinese Academy of Sciences, Beijing 100190, China; wuwlong924@foxmail.com (P.W.); whzhu@mail.ie.ac.cn (W.Z.); fyq@mail.ie.ac.cn (Y.F.); gyfang@mail.ie.ac.cn (G.F.); 2 University of Chinese Academy of Sciences, Beijing 100049, China; 3 Institute of Electronics, Chinese Academy of Sciences, Beijing 100190, China

**Keywords:** aeromagnetic compensation, optically pumped magnetometer, transfer function, coherent noise suppression, referenced magnetometer

## Abstract

An aeromagnetic survey is an important method in magnetic anomaly detection and geophysical prospecting. The magnetic field is typically measured by optically pumped magnetometers (OPM) installed on the aircraft. The measurement accuracy of the OPM is easily affected by the platform-generated magnetic fields. Therefore, aeromagnetic compensation is necessary. The traditional compensation model only considers the permanent, induced, and eddy current interference magnetic field of the aircraft platform. However, the interference field produced by the avionics system, and the relative motion between the aircraft and the magnetometer, are still not taken into account. To address this issue, we proposed a novel strategy to eliminate the additional interference of the platform with two OPMs. Among them, the OPM located farther away from the aircraft serves as a sensing magnetometer, whereas the near OPM serves as a reference magnetometer. The coherent noise suppression method is used to process the residual magnetic field interference after compensation. By establishing the interference magnetic transfer function between the two sensors, the interference field can be suppressed. The results of the experiments demonstrate the effectiveness of the novel strategy, and the standard deviation of residual interference drops from 0.065 nT to 0.045 nT.

## 1. Introduction

Aeromagnetic exploration is an important method used in geophysical prospecting, especially in mineral exploitation, underground unexploded ordnance detection, and underwater target detection [1,2,3,4]. Compared with surface and satellite magnetic surveys, aeromagnetic exploration has huge advantages in terms of resolution, efficiency, and security [5,6]. In addition, the field measured by an optically pumped magnetometer (OPM) is typically reliable and accurate [7]. More and more attention has been paid to its application in recent years. The platform for aeromagnetic exploration is an aircraft like a helicopter or fixed-wing aircraft. However, the construction components of the aircraft always contain some ferromagnetic materials, which will have a severe impact on aeromagnetic measurements of the total-field and degrade the measurement accuracy of the OPM. Therefore, the removal of the aircraft interference magnetic field is necessary for the aeromagnetic surveys.

Aeromagnetic compensation research can be traced back to World War II, when it was used for detecting submarines under the water. After the war, this technology was widely used in the civilian field, such as geological surveys and geophysical exploration. Given that the aircraft platform would affect the accuracy of OPM measurement, W.E. Tolles proposed a specific compensation model in 1940s, and divided the aircraft’s interference field into three parts: the permanent interference magnetic field, induced interference magnetic field, and eddy current induced interference magnetic field [8,9]. In 1961, Paul Leliak proposed sinusoidal maneuvers to improve the compensation performance [10]. In 1980, B.W. Leach proposed a ridge regression algorithm to solve multi-collinearity in the aeromagnetic compensation model, and experimental results show that this method performed well in improving compensation performance [11]. In 1995, Hardwicke designed a gradient magnetic measurement system with a fixed-wing platform, which was the innovative three-axis aeromagnetic gradiometry compensation [12]. Since then, various aviation magnetic field compensation methods and models have been reported [13,14,15,16,17]. Around 2005, Nelson carried out a series of experiments with an experimental flight system, including the evaluation of noise levels during low-altitude flights, prediction of airborne noise levels with ground measurements, and assessment of the noise levels of an aircraft’s components [18,19]. Among the methods, both the FOM and the signal’s standard deviation could be used as evaluation criteria for compensation performance. In 2012, M. Woloszyn established the aircraft’s interfering magnetic field through the finite element method (FEM) and used the least squares method to compensate for the aircraft’s interfering magnetic field [20]. G. Noriega studied the stability of aeromagnetic compensation and the compensation of aeromagnetic gradient data from 2011 to 2014 and gave a numerical method for effectively evaluating the reliability of calibration flight results [21,22]. In recent years, Canadian RMS Company systematically demonstrated the effectiveness and proved the viability and reliability of using the standard deviation of the residual interfering signal as an assessment criterion [23]. Besides, some researchers incorporated other factors into the compensation model, and improved the compensation accuracy [24].

At present, aeromagnetic compensation is predominantly based on the conventional aeromagnetic compensation model due to its excellent compensation performance. However, the model can only process the permanent, induced, and eddy current interference magnetic field of the platform. The interference magnetic field produced by the relative motion between the flight and the OPMs and the flight electronic systems are still not taken into account. In this paper, we propose a novel strategy for processing the additional interference field with the coherent noise suppression (CNS) method. The results of the experiments demonstrate the validity of the novel strategy for improving the aeromagnetic compensation performance.

## 2. Aeromagnetic Compensation Model

According to the classical aeromagnetic compensation Tolles-Lawson model [8,9], the aircraft interfering sources can be divided into three types: the permanent magnetic field, induced magnetic field, and eddy current magnetic field.

In order to compensate for the platform-generated magnetic fields, in the aeromagnetic compensation model, a three-axis magnetometer is used to measure the attitude of the platform. We defined the quantities *T*, *L*, and *V* as the three component fields of the geomagnetic vector, and obtained the magnitude of geomagnetic field *He*. The direction cosines of the angles between the geomagnetic field and the platform are denoted as $u_{1}$, $u_{2}$, and $u_{3}$, and they can be expressed as:

(1) $$\begin{array}{l} u_{1}=T\left(t\right)/H_{e}\left(t\right)=T\left(t\right)/\sqrt{T{\left(t\right)}^{2}+L{\left(t\right)}^{2}+V{\left(t\right)}^{2}}\\ u_{2}=L\left(t\right)/H_{e}\left(t\right)=L\left(t\right)/\sqrt{T{\left(t\right)}^{2}+L{\left(t\right)}^{2}+V{\left(t\right)}^{2}}\\ u_{3}=V\left(t\right)/H_{e}\left(t\right)=V\left(t\right)/\sqrt{T{\left(t\right)}^{2}+L{\left(t\right)}^{2}+V{\left(t\right)}^{2}} \end{array}$$

Define $H_{T}\left(t\right)$ as the total intensity of the interference field measured by the total field magnetometer. This quantity can be decomposed into permanent, induced, and eddy-current sources. Thus,

(2) $$H_{T}\left(t\right)=H_{Perm}\left(t\right)+H_{Ind}\left(t\right)+H_{Eddy}\left(t\right)$$

The permanent source interference magnetic field $H_{Perm}\left(t\right)$ is the permanent magnetism of the various ferromagnetic parts of the aircraft. It has the following mathematical expression:

(3) $$H_{Perm}\left(t\right)=c_{1}u_{1}+c_{2}u_{2}+c_{3}u_{3}=\sum_{i=1}^{3}A_{i}\left(t\right)c_{i}$$

The induced interference magnetic field $H_{Ind}\left(t\right)$ is created in the soft iron parts by the earth’s magnetic field. It can be expressed as:

(4) $$H_{Ind}\left(t\right)=H_{e}\left(t\right)\left(c_{4}u_{1}^{2}+c_{5}u_{1}u_{2}+c_{6}u_{1}u_{3}+c_{7}u_{2}^{2}+c_{8}u_{2}u_{3}+c_{9}u_{3}^{2}\right)=\sum_{i=4}^{9}A_{i}\left(t\right)c_{i}$$

As the aircraft manoeuvres in the earth’s magnetic field, the eddy current is produced by different metal components, which is proportional to the time change rate of the flux through these components. The eddy current produces a magnetic field in the direction perpendicular to the plane of the conducting surface. The eddy-current interference field can be expressed as:

(5) $$H_{Eddy}\left(t\right)=H_{e}\left(t\right)\left(\begin{array}{l} c_{10}u_{1}u_{1}^{\prime }+c_{11}u_{1}u_{2}^{\prime }+c_{12}u_{1}u_{3}^{\prime }+c_{13}u_{2}u_{1}^{\prime }+c_{14}u_{2}u_{2}^{\prime }\\ +c_{15}u_{2}u_{3}^{\prime }+c_{16}u_{3}u_{1}^{\prime }+c_{17}u_{3}u_{2}^{\prime }+c_{18}u_{3}u_{3}^{\prime } \end{array}\right)=\sum_{i=10}^{18}A_{i}\left(t\right)c_{i}$$

The coefficients $c_{i},i=1,2,\cdots ,18$ represent the compensation parameter, which are assumed to be consistent for a stable given aircraft configuration. Assuming these coefficients were known exactly, one would have an explicit mathematical model for predicting the aircraft interference field. Subtracting the calculated $H_{T}\left(t\right)$ from the measured signal, the aircraft interference magnetic field would be eliminated. The time-varying quantities $A_{i}\left(t\right),i=1,2,\cdots ,18$ can be calculated from knowledge of $u_{1},u_{2},u_{3}$ and their time derivatives $u_{1}^{\prime },u_{2}^{\prime },u_{3}^{\prime }$. Therefore, the platform-generated magnetic fields on the OPM can be expressed as:

(6) $$H_{T}\left(t\right)=\sum_{i=1}^{18}A_{i}\left(t\right)c_{i}$$

The corresponding matrix notation can be expressed as:

(7) H=AC

where H is a column vector constituted by the sampling points of $H_{T}\left(t\right)$, and it can be expressed as $H={\left[\begin{array}{cccc} H\left(1\right) & H\left(2\right) & \cdots & H\left(n\right) \end{array}\right]}^{T}$; C is a column vector constituted by $c_{i},i=1,\cdots ,18$, and it can be expressed as $C={\left[\begin{array}{cccc} c_{1} & c_{2} & \cdots & c_{18} \end{array}\right]}^{T}$; and A is the corresponding matrix constituted by the sampling points of attitude item, and it can be expressed as:

(8) $$A=\left[\begin{array}{cccc} A_{1}\left(1\right) & A_{2}\left(1\right) & \cdots & A_{18}\left(1\right)\\ A_{1}\left(2\right) & A_{2}\left(2\right) & \cdots & A_{18}\left(2\right)\\ \vdots & \vdots & \ddots & \cdots \\ A_{1}\left(n\right) & A_{2}\left(n\right) & \cdots & A_{18}\left(n\right) \end{array}\right]$$

The compensation parameters can be obtained by the least squares solution from Equation (7). It can be expressed as:

(9) $$C_{LS}={\left(A^{T}A\right)}^{-1}A^{T}H$$

Although equations define 18 separate terms to describe the complete aircraft interference model, normally two of the terms are eliminated on the basis of redundancy stemming from certain assumptions [11]. They can be expressed as follows:

(10) $$u_{1}^{2}+u_{2}^{2}+u_{3}^{2}=1$$

(11) $$u_{1}u_{1}^{\prime }+u_{2}u_{2}^{\prime }+u_{3}u_{3}^{\prime }=0$$

Because multi-collinearity existed in the model, the inverse matrix of $A^{T}A$ does not exist, theoretically. However, the measurement noise from the sensor and the geomagnetic environment will affect the results of fluxgate, which means that the least squares method still has some operability in the actual operation. Still, the result of least squares is still not reliable enough. Ridge regression (RR) analysis is an effective biased estimation technique, which is specially aimed at the problem of multi-collinearity in a regression model.

The solution of equation by RR analysis can be expressed as:

(12) $$C_{rr}={\left(A^{T}A+kI\right)}^{-1}A^{T}H$$

where *k* is a ridge parameter and I is the identity matrix.

Compared with Equation (9) in aeromagnetic compensation, Equation (12) leads to better compensation results [11]. In a typical case, when using Equation (12) in a calibration flight, the compensation coefficients can be obtained. Then, by bringing the obtained compensation coefficients back into the equation, the magnetic fields generated by the platform can be eliminated. It should be noted that if the aircraft undergoes a feature change due to engine or mechanical damage after calibration, it should be recalibrated before the next aeromagnetic survey.

## 3. The Coherent Noise Suppression Method

**Figure 1:**
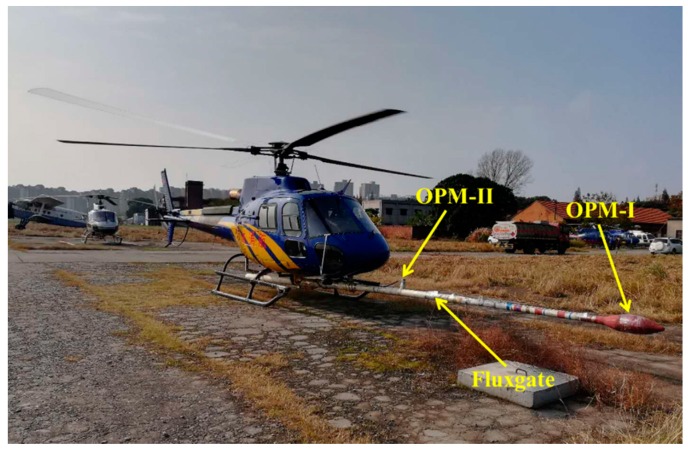
The helicopter platform and equipment.

The coherent noise suppression is a practical method for the noise cancellation method. The spatial coherence of the interference field indicates that a noise cancellation system based on data from reference magnetometers would offer better noise reduction than other approaches. The strategy for improving the compensation performance is by two sensors which are installed at different locations of a helicopter, as is shown in Figure 1.

**Figure 2:**
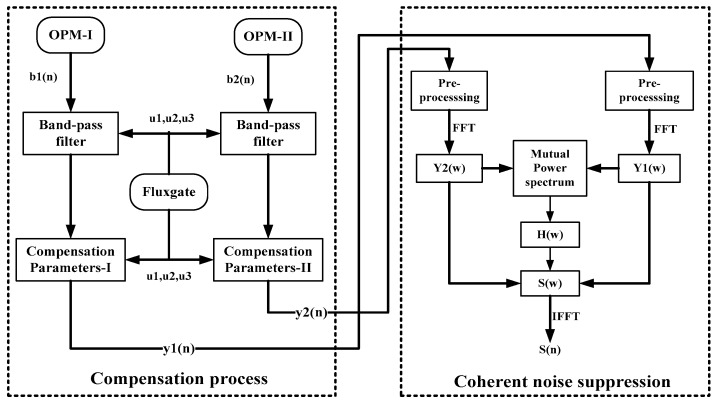
Block diagram of the aeromagnetic compensation and coherent noise suppression algorithm.

As shown in Figure 1, the optically pumped magnetometer I (OPM-I) acts as a sensing magnetometer, while the optically pumped magnetometer II (OPM-II) acts as the reference magnetometer. The structure of the aeromagnetic compensation and coherent noise suppression algorithm is shown in Figure 2.

### 3.1. The Mathematical Model

Both of the two magnetometers measure the same geomagnetic field, which is the magnetic field we need. At the same time, the interference magnetic field is also measured by two magnetometers; what is different is that the magnetometer closer to the helicopter sustains bigger interference than the farther one. In addition, the interference field of the two magnetometers has a high coherence. Through the calibration flight, we get the two sets of compensation parameters separately for the OPM-I and OPM-II. After the compensation, we can obtain magnetic field data sequences $y_{1}\left(n\right)$ and $y_{2}\left(n\right)$. The $y_{1}\left(n\right)$ involves the signal field s(n) and interference field e(n), while $y_{2}\left(n\right)$ involves the signal field s(n) and stronger interference field $e_{2}\left(n\right)$. The relationship of these records can be expressed as:

(13) $$\begin{array}{l} y_{1}(n)=s(n)+e(n)+w_{1}\left(n\right)\\ y_{2}(n)=s(n)+e_{2}(n)+w_{2}\left(n\right) \end{array}$$

The *n* denotes the sample-index, where $w_{1}\left(n\right)$ and $w_{2}\left(n\right)$ represent the intrinsic noises of OPM-I and OPM-II, respectively. Nevertheless, the signal magnetic field s(n) includes a strong DC component. Thus, the bias from s(n) should be removed during the processing and might be recovered afterward by adding the value of the removed bias to the s(n). Because of the coherence of interference field, the interference field e(n) and $e_{2}\left(n\right)$ can be expressed as:

(14) $$e_{2}\left(n\right)=h(n)*e(n)$$

where h(n) is the transfer function.

### 3.2. The Proposed Coherent Noise Suppression Method

We consider the detector as a 2 × 2 linear time invariant system and then propose a coherent noise suppression method, which can suppress the residual interference field from the helicopter and improve the aeromagnetic compensation performance based on traditional aeromagnetic compensation.

After applying a discrete Fourier transform (DFT) on both sides of Equation (13), they can be represented in the frequency domain as:

(15) $$\begin{array}{l} Y_{1}\left(\omega \right)=S\left(\omega \right)+E\left(\omega \right)+W_{1}\left(\omega \right)\\ Y_{2}\left(\omega \right)=S\left(\omega \right)+H\left(\omega \right)\cdot E\left(\omega \right)+W_{2}\left(\omega \right) \end{array}$$

Notice that the intrinsic noise power of the magnetometers is much smaller than the interference field. For the sake of simplification, we ignore the influence of $w_{1}\left(n\right)$ and $w_{2}\left(n\right)$. Hence, the auto-spectra and cross-spectra of $Y_{1}\left(\omega \right)$ and $Y_{2}\left(\omega \right)$ are shown as follows:

(16) $$\begin{array}{l} P_{Y_{1}Y_{1}}\left(\omega \right)=P_{SS}\left(\omega \right)+P_{EE}\left(\omega \right)\\ P_{Y_{1}Y_{2}}\left(\omega \right)=P_{SS}\left(\omega \right)+H^{*}\left(\omega \right)P_{EE}\left(\omega \right)\\ P_{Y_{2}Y_{1}}\left(\omega \right)=P_{SS}\left(\omega \right)+H\left(\omega \right)P_{EE}\left(\omega \right)\\ P_{Y_{2}Y_{2}}\left(\omega \right)=P_{SS}\left(\omega \right)+H\left(\omega \right)H^{*}\left(\omega \right)P_{EE}\left(\omega \right) \end{array}$$

where $P_{SS}\left(\omega \right)$ and $P_{EE}\left(\omega \right)$ are the auto-spectra of the signal and interference field, and H(ω) is the discrete Fourier transform of h(n). Then, the transfer function H(ω) can be described as:

(17) $$H\left(\omega \right)=\frac{P_{Y_{2}Y_{2}}\left(\omega \right)-P_{Y_{2}Y_{1}}\left(\omega \right)}{P_{Y_{1}Y_{2}}\left(\omega \right)-P_{Y_{1}Y_{1}}\left(\omega \right)}$$

Then, the s(n) can be reconstructed from the frequency domain by the inverse Fourier transform:

(18) $$s\left(n\right)=F^{-1}\left(y_{1}\left(\omega \right)-\frac{y_{2}\left(\omega \right)-y_{1}\left(\omega \right)}{H\left(\omega \right)-1}\right)$$

## 4. Results and Discussion

### 4.1. The Compensation and Test Flight

The experiment is designed to carry out aeromagnetic compensation using two OPMs mounted on the helicopter. The fluxgate magnetometer is used to measure the attitude of the platform. In the experiment, the magnetic field is measured by an OPM-I at the end of the bar, which is the sentinel magnetometer. The OPM-II is fixed at the middle of the bar, which is the reference magnetometer. The OPM-I is the Cs Optical pump magnetometer which is made by the IECAS, the OPM-II is CS-3, and the fluxgate magnetometer model is TFM-100G2. Both of the OPMs have the same performance with a sensitivity of 0.6 pT/√Hz@1 Hz [25]. The type of data recording system is AADC510, which is manufactured by RMS Instruments [26]. The sampling rate of the system is 40 Hz.

**Figure 3:**
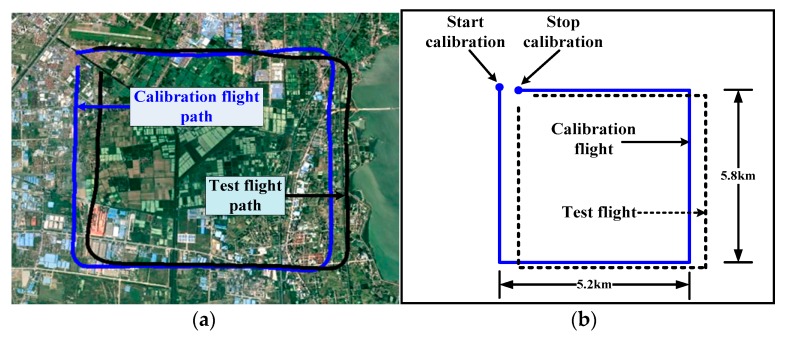
Calibration and test flight path in google earth (**a**) and its scale drawing (**b**).

The flight path used for the compensation flight and test flight is shown in Figure 3.

In order to avoid being affected by the magnetic interference of the ground, the experimental height was about 3000 m. First, the helicopter flew a calibration box, and then a test box was flown. These two flight boxes are very similar. Both of them contain four orthogonal magnetic headings, and each line of the boxes contains calibration maneuvers consisting of about ±10° rolls, ±5° pitches and ±5° yaws. In an ideal calibration flight, the calibration box and the test box are the same shape. However, in a practical flight, it is difficult to keep these two boxes in the same shape. The calibration flight is used to obtain the compensation parameters, and the test flight is used to test the validation of the compensation parameters.

### 4.2. Compensation Parameters and Compensation Results

**Figure 4:**
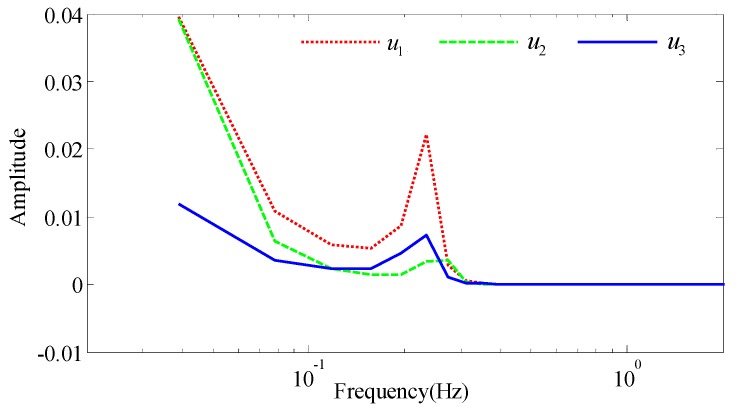
The power spectral density of the direction cosines.

A fluxgate magnetometer is used to obtain the attitude of the helicopter. In order to better obtain the maneuvering characteristics of the aircraft, the power spectral density of the direction cosines $u_{1}$, $u_{2}$, and $u_{3}$ is shown in Figure 4. From the figure, the aircraft maneuver frequency is mainly between 0.15 Hz and 0.4 Hz during the compensation flight. By designing a responsive high-pass filter, we can get the optimal interference field and attitude information for the compensation parameters regression.

The compensation parameters of the OPM-I and OPM-II are separately listed in Table 1 and Table 2. It can be clearly seen from the Tables that the compensation parameters of the OPM-II are larger than those of OPM-I, which is consistent with the real situation.

**Figure 5:**
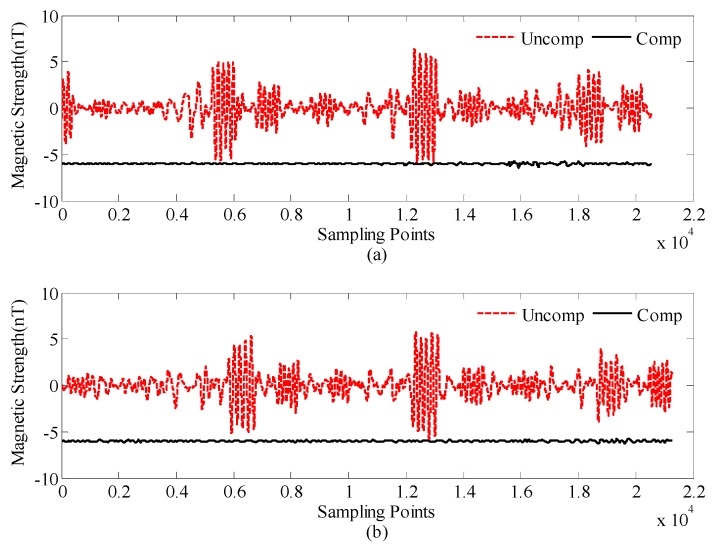
Compensation results of OPM-I: (**a**) calibration flight data and (**b**) test flight data.

Figure 5 shows the OPM-I compensation results of the calibration and test flight data, by using the compensation parameters of Table 1. The red dashed line is the uncompensated data of OPM-I, which is the original data processed by a filter. The solid line is the compensated data of OPM-I, which is offset by 1 nT for clarity.

**Figure 6:**
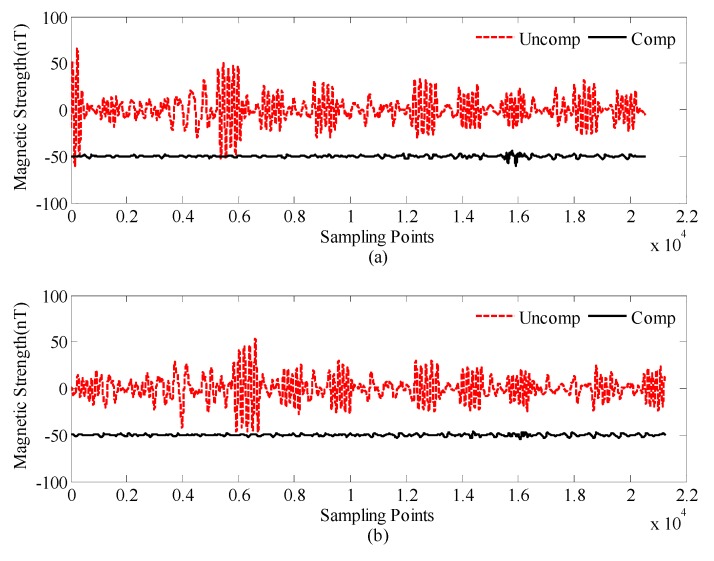
Compensation results of OPM-II: (**a**) calibration flight data and (**b**) test flight data.

Figure 6 shows the OPM-II compensation results of the calibration and test flight data, by using the optimal compensation parameters of Table 2. The red dashed line is the uncompensated data of OPM-II, which is the original data processed by a filter. The solid line is the compensated data of OPM-II, which is offset by 1 nT for clarity.

The standard deviation and improvement ratio (IR) are used to assess the results of the compensation performance. The IR is the ratio of the standard deviation of the raw signal to that of the compensated signal. The quantitative comparisons of two OPMs are presented in Table 3.

Table 3 shows that both of the compensation parameters can compensate for the helicopter interference well. The compensation method and the choice of filter are reasonable. From Table 3, it can be seen that the standard deviation of the residual interference field OPM-I and OPM-II is 0.065 nT and 1.16 nT, respectively. The much bigger interference of OPM-II can originate from the closer distance to the helicopter, which suppressed the bigger non-maneuver interference.

### 4.3. The Results and Discussion of the Proposed CNS Method

A. Results of the proposed CNS method

**Figure 7:**
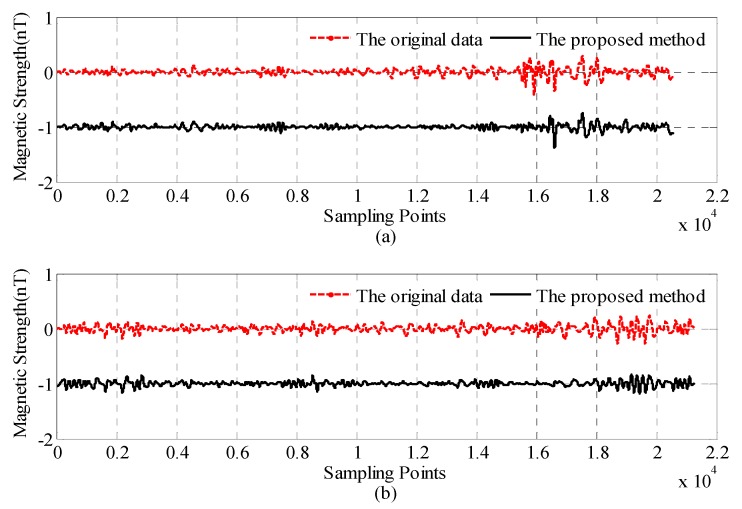
The interference field before and after the CNS method: (**a**) calibration flight and (**b**) test flight.

The magnetic field after compensation and the CNS method are shown in Figure 7. The red dashed line is the compensated field before the proposed CNS method. The solid line is the compensated data after the proposed CNS method, which is offset by 1 nT for clarity.

The quantitative comparisons of before and after the proposed CNS method are presented in Table 4. The proposed CNS method can improve the compensation performance by about 30% compared to the traditional method.

As you can see from Figure 7 and Table 4, part of the interference field of the residual interference of the helicopter is eliminated by the proposed CNS method, and there is still about 0.045 nT and 0.046 nT residual interference field for the calibration and test flight.

B. Analysis of the results

From Table 4, it can be found that about 0.045 nT residual interference field has not been eliminated after the CNS method. It is necessary to analyze the composition of the remaining interference field. The remaining interference field may include the geomagnetic background and the noise of the OPM, the geomagnetic gradients coupled to the movement of the platform, and the un-complete parts of the residual interference field of the helicopter. The three parts will be described separately as follows: 

(1) The geomagnetic background and the noise of the OPM

**Figure 8:**
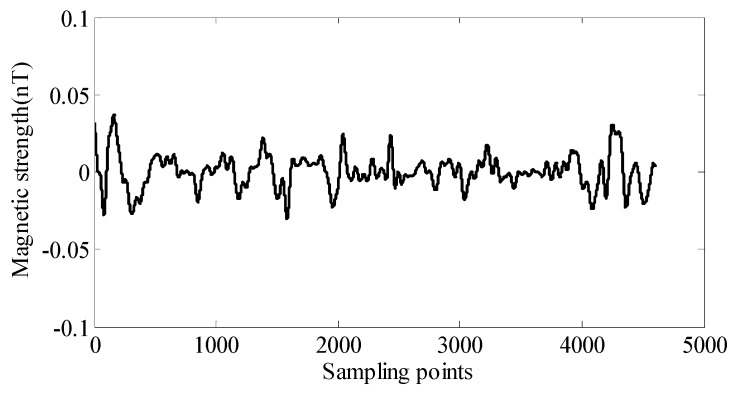
The background geomagnetic interference field.

Before the aeromagnetic flight test, the background magnetic field has been obtained through the ground background noise test, which is shown in Figure 8. The level of the geomagnetic background field including the noise of the OPM is about 0.0108 nT. This is much smaller than the residual interference field, so it is not the main component of the remaining interference field.

(2) The geomagnetic gradients coupled to the movement of the platform

**Figure 9:**
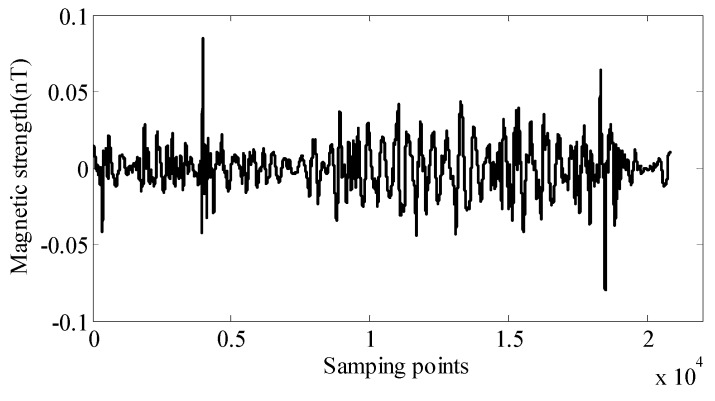
The geomagnetic gradients interference field.

According to the International Geomagnetic Reference Field (IGRF) model and the trajectory of the aircraft by the GPS, the interference field of the geomagnetic gradients coupled to the movement of the platform is shown in Figure 9. The level of the geomagnetic gradients interference is about 0.0144 nT, which is also smaller than the residual interference field, so it is also not the main reason for forming the remaining interference field.

(3) The correlation of the OPM-I and OPM-II interference field

**Figure 10:**
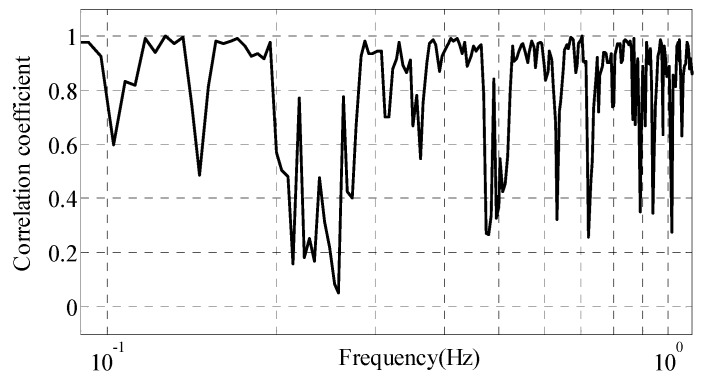
The correlation coefficient of the interference field in OPM-I and OPM-II.

The correlation coefficient of the OPM-I and OPM-II interference magnetic field is shown in Figure 10. From the figure, the correlation coefficient is high and can reach or exceed 0.9 in the majority of the frequency band from 0.1 Hz to 1 Hz. However, it also shows poor correlation in some frequency bands, like 0.2–0.28 Hz, 0.46–0.52 Hz, and other frequency points. As we know, the different frequency bands’ interference fields likely correspond to the helicopter’s different parts. According to the near-field attenuation characteristics of the magnetic interference sources, the partial differences between the OPM-I and OPM-II interference fields are also normal. The CNS method would have a good suppression effect for a highly correlated interference field, but it has little effect on poorly correlated parts. Therefore, the irrelevant part of the interference field is most likely to be a residual interference field after the CNS method.

### 4.4. The Magnetic Anomaly Detection Experiment

**Figure 11:**
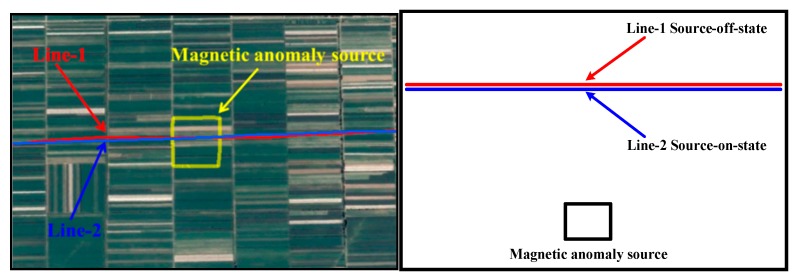
The flight path in Google Earth and its scale drawing.

In order to test the validity of the proposed method, we have designed the magnetic anomaly detection experiment. The magnetic anomaly signal is produced by a square closed coil, whose center coordinate is about 30°23′11′′ N, 111°56′53.2′′ E. The length of the square coil is about 200 m and the excitation current is about 14.5 A. The coil and flight lines are shown in Figure 11. The flight test consists of two test lines, while the line-1 is ongoing in the source shutdown state and the line-2 is in the source boot state. The flight height of the two lines is about 300 m.

**Figure 12:**
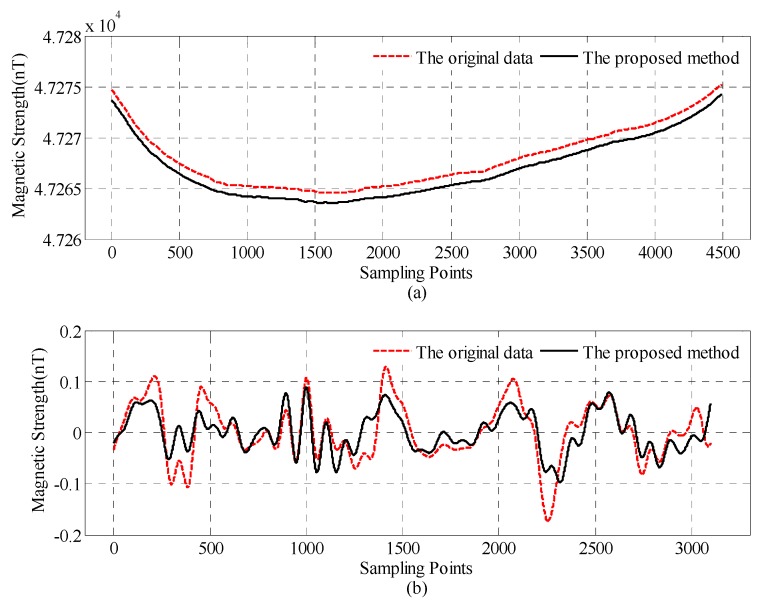
The magnetic field of the flight survey line-1: (**a**) total magnetic field and (**b**) interference magnetic field.

Figure 12a shows the total magnetic field of the line-1, where the red dashed line is the compensated data before the proposed CNS method. The solid line is the compensated data after the proposed CNS method, which subtracts 1 nT for clarity. Figure 12b shows the band pass magnetic field of the line-1, where the red dashed line is the compensated data before the proposed CNS method. The solid line is the compensated data after the proposed CNS method.

Standard deviation is used to assess the results of the proposed method. The quantitative comparisons of before and after the proposed CNS method are presented in Table 5.

As you can see from Figure 12 and Table 5, after the proposed CNS method, the interference field standard deviation is suppressed from 0.0555 nT to 0.039 nT, and the peak to peak value is reduced from 0.297 nT to 0.184 nT.

**Figure 13:**
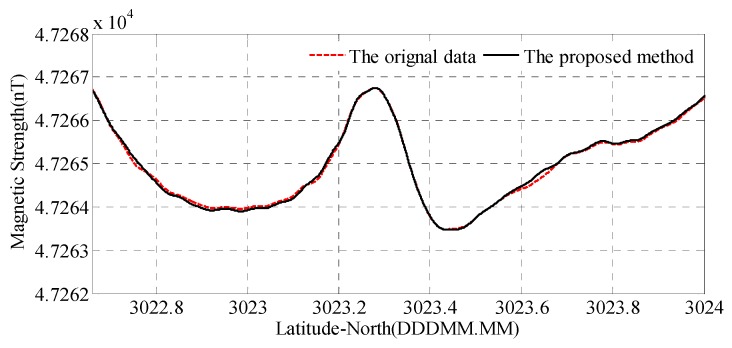
The magnetic field of the flight survey line-2.

Figure 13 shows the total magnetic field of the line-2, where the red dashed line is the compensated data before the proposed CNS method. The solid line is the compensated data after the proposed CNS method. From the figure, the magnetic anomaly signal can be clearly seen. The only difference between the signal before and after processing is that the processed signal is smoother than the original. The results indicate that validity of the proposed method.

## 5. Further Investigation

In this paper, we attempt to use the reference magnetometer to eliminate the helicopter’s interference magnetic field. The results indicate the effectiveness of the proposed method. However, there are still some interfering magnetic fields that have not been eliminated, which are considered to be the uncorrelated interference magnetic field of OPM-I and OPM-II.

In future work, we expect to optimize the system by increasing the number of the reference magnetometers and optimizing the position of the reference sensors. Then, the correlation of the magnetometer interference field will be further increased and the aeromagnetic compensation performance will continue to be improved. In addition, the method in this article applies in principle to signals in other frequency bands, such as extremely low frequency (ELF) signals. We hope to gain a performance improvement of the entire signal band through this method.

## 6. Conclusions

The traditional aeromagnetic compensation model considers the permanent, induced, and eddy currents interference magnetic field of the helicopter, and the quality of the aeromagnetic data is significantly improved after compensation. However, the interference fields from helicopter engine systems, avionics systems, and other interference sources were not considered in traditional models. These interference sources still affect the quality of aeromagnetic data, making it difficult to detect weak magnetic signals. To address this issue, the CNS method is proposed to suppress the magnetic interference after the traditional compensation with an auxiliary reference magnetometer. When the compensation flight is completed, we obtained the compensation parameters, and then used the CNS method to obtain the interference transfer function between the two OPMs. Through testing and survey flights, the effectiveness of the proposed method is verified and the standard deviation of the residual interference field dropped from 0.065 nT to 0.045 nT. In addition, the source of the residual interference magnetic field is also analyzed in this paper. At the end of the paper, the system optimization method is proposed to further enhance the performance of the aeromagnetic compensation system.

## Figures and Tables

**Table 1:** Compensation parameters of OPM-I.

Parameter	Value	Parameter	Value	Parameter	Value
c1	32.5	c2	4.24	c3	7.84
c4	5.90 × 10^−3^	c5	−2.71 × 10^−6^	c6	−2.09 × 10^−5^
c7	6.20 × 10^−3^	c8	1.74 × 10^−4^	c9	5.80 × 10^−3^
c10	8.28 × 10^−4^	c11	−7.46 × 10^−5^	c12	−1.84 × 10^−5^
c13	5.42 × 10^−5^	c14	6.72 × 10^−4^	c15	−6.75 × 10^−5^
c16	−6.57 × 10^−5^	c17	−1.65 × 10^−5^	c18	8.69 × 10^−4^

**Table 2:** Compensation parameters of OPM-II.

Parameter	Value	Parameter	Value	Parameter	Value
c1	279.3	c2	109.1	c3	−196.9
c4	0.0767	c5	−8.11 × 10^−5^	c6	−1.75 × 10^−4^
c7	0.080	c8	1.95 × 10^−3^	c9	0.0766
c10	8.90 × 10^−3^	c11	−1.20 × 10^−3^	c12	1.56 × 10^−4^
c13	3.09 × 10^−4^	c14	8.20 × 10^−3^	c15	−8.68 × 10^−4^
c16	−2.10 × 10^−4^	c17	3.89 × 10^−4^	c18	9.50 × 10^−3^

**Table 3:** Standard deviation and IR of different OPMs.

Magnetometer	Flight	Uncompensated (nT)	Compensated (nT)	IR
OPM-I	Calibration	1.5305	0.0649	23.6
	test	1.4172	0.0654	21.67
OPM-II	Calibration	13.8061	1.2283	11.24
	test	12.3317	1.1055	11.15

**Table 4:** Standard deviation and IR of the proposed CNS method.

Magnetometer	Flight	Uncompensated (nT)	The CNS method (nT)	IR
OPM-I	Calibration	1.5305	0.0453	33.78
	Test	1.4172	0.0460	30.80

**Table 5:** Standard deviation and peak to peak value of the flight survey line-1.

Method	Standard Deviation (nT)	Peak to Peak Value (nT)
Original data	0.0555	0.297
CNS method	0.0390	0.184

## References

[1] Nabighian M.N., Grauch V.J.S., Hansen R.O., LaFehr T.R., Li Y., Peirce J.W., Phillips J.D., Ruder M.E. (2005). The historical development of the magnetic method in exploration. Geophysics.

[2] Beard L.P., Doll W.E., Gamey T.J., Scott Holladay J., Lee J.L.C., Eklund N.W., Sheehan J.R., Norton J. (2008). Comparison of Performance of Airborne Magnetic and Transient Electromagnetic Systems for Ordnance Detection and Mapping. J. Environ. Eng. Geophys..

[3] Hood P. (2007). History of aeromagnetic surveying in Canada. Lead. Edge.

[4] Doll W.E., Gamey T.J., Bell D.T., Beard L.P., Sheehan J.R., Norton J., Holladay J.S., Lee J.L.C. (2012). Historical development and performance of airborne magnetic and electromagnetic systems for mapping and detection of unexploded ordnance. J. Environ. Eng. Geophys..

[5] Zhang C.D. (2003). The Past, Present and Future of the Satellite Magnetic Survey. Geophys. Geochem. Explor..

[6] Olsen N., Hulot G., Sabaka T.J. (2010). Measuring the Earth’s Magnetic Field from Space: Concepts of Past, Present and Future Missions. Space Sci. Rev..

[7] Hardwick C.D. (1984). Non-oriented cesium sensors for airborne magnetometry and gradiometry. Explor. Geophys..

[8] Tolles W.E., Mineola N.Y. (1954). Compensation of Aircraft Magnetic Fields. U.S. Patent.

[9] Tolles W.E., Mineola N.Y. (1955). Magnetic Field Compensation System. U.S. Patent.

[10] Leliak P. (1961). Identification and evaluation of magnetic-field sources of magnetic airborne detector equipped aircraft. IRE Trans. Aerosp. Navig. Electron..

[11] Leach B.W. (1980). Aeromagnetic compensation as a linear regression problem. Information Linkage between Applied Mathematics and Industry.

[12] Hardwick C.D. (1996). Aeromagnetic gradiometry in 1995. Explor. Geophys..

[13] Zhang B., Guo Z., Qiao Y. A simplified aeromagnetic compensation model for low magnetism UAV platform. Proceedings of the IEEE International Geoscience and Remote Sensing Symposium (IGARSS).

[14] Williams P.M. (1993). Aeromagnetic compensation using neural networks. Neural Comput. Appl..

[15] Deng P., Lin C., Tan B., Jian Z. Application of adaptive filtering algorithm based on wavelet transformation in aeromagnetic survey. Proceedings of the 3rd IEEE International Conference on Computer Science and Information Technology (ICCSIT).

[16] Lin C., Zhou J., Yang Z. (2015). A method to solve the aircraft magnetic field model basing on geomagnetic environment simulation. J. Magn. Magn. Mater..

[17] Dou Z., Ren K., Han Q., Niu X. A Novel Real-Time Aeromagnetic Compensation Method Based on RLSQ. Proceedings of the 2014 Tenth International Conference on Intelligent Information Hiding and Multimedia Signal Processing (IIH-MSP).

[18] Nelson J.B. (2002). Aeromagnetic Noise during Low-Altitude Flights over the Scotian Shelf.

[19] Nelson J.B. (2001). Predicting In-Flight Mad Noise from Ground Measurements.

[20] Woloszyn M. (2012). Analysis of aircraft magnetic interference. Int. J. Appl. Electromagn. Mech..

[21] Noriega G. (2013). Model stability and robustness in aeromagnetic compensation. First Break.

[22] Noriega G. (2011). Performance measures in aeromagnetic compensation. Lead. Edge.

[23] Dou Z., Han Q., Niu X., Peng X., Guo H. (2016). An Adaptive Filter for Aeromagnetic Compensation Based on Wavelet Multiresolution Analysis. IEEE Geosci. Remote Sens..

[24] Han Q., Dou Z., Tong X., Peng X., Guo H. (2017). A Modified Tolles-Lawson Model Robust to the Errors of the Three-Axis Strapdown Magnetometer. IEEE Geosci. Remote Sens..

[25] The Brochure of CS-3 High Sensitivity CS Magnetometer Sensor. https://scintrexltd.com/wp-content/uploads/2017/03/CS-3-Brochure-762711_3.pdf.

[26] The Description of AARC510 Adaptive Aeromagnetic Real-Time Compensator. https://2117-ca.all.biz/aarc510-adaptive-aeromagnetic-real-time-g3383.

